# Solitary pouch ulcer syndrome—a newly recognized phenotype of the ileal pouch disorders

**DOI:** 10.1093/gastro/goae073

**Published:** 2024-07-08

**Authors:** Bo Shen, Huai-Bin Mabel Ko, Hong Ma, Ravi Kiran, James Church

**Affiliations:** The Global Integrated Center for Colorectal Surgery and IBD Interventional Endoscopy, Columbia University Irving Medical Center/New York-Presbyterian Hospital, New York, NY, USA; Department of Pathology and Cell Biology, Columbia University Irving Medical Center/New York Presbyterian Hospital, New York, NY, USA; Division of Abdominal Radiology, Columbia University Irving Medical Center/New York Presbyterian Hospital, New York, NY, USA; The Global Integrated Center for Colorectal Surgery and IBD Interventional Endoscopy, Columbia University Irving Medical Center/New York-Presbyterian Hospital, New York, NY, USA; The Global Integrated Center for Colorectal Surgery and IBD Interventional Endoscopy, Columbia University Irving Medical Center/New York-Presbyterian Hospital, New York, NY, USA

## Introduction

Pouchitis is the most common long-term sequela after restorative proctocolectomy and ileal pouch–anal anastomosis (IPAA) for ulcerative colitis [1]. The patient with pouchitis commonly presented with frequent bowel movements, abdominal pain, urgency, and rarely bleeding. Most patients with initial episodes of pouchitis respond favorably to oral antibiotics. However, some patients required maintenance therapy of antibiotics to keep the symptoms in remission. Clinically, we have noticed that stool frequency, urgency, antibiotic dependency, and sometimes bleeding in some patients result from dyschezia and incomplete evacuation from structural or functional outlet obstruction. Here, we described a representative case from our Pouch Center in which the patient presents with dyschezia, frequent stool, solitary ulcer at the distal pouch fold, and abnormal anopouch manometry.

## Case report

A 28-year-old male presented to our Pouch Center with chief complaints of frequent stools, excessive straining, and incomplete evacuation with a diagnosis of chronic antibiotic-dependent pouchitis. He was diagnosed with ulcerative colitis at age 20 and underwent restorative colectomy and two-stage J-IPAA for medical refractory disease to infliximab, adalimumab, vedolizumab, and tofacitinib. His postoperative course was complicated with partial small bowel obstruction and anastomotic leak, which was drained via interventional radiology. His previous magnetic resonance enterography showed a distended pouch with mild wall thickening and postcontrast enhancement likely from “pouchitis.” There were chronically dilated small bowel loops without evidence of intestinal obstruction.

Ever since the stoma closure, he experienced the presenting symptoms and gradually got worse. His symptoms required frequent and then persistent use of oral antibiotics (ciprofloxacin, metronidazole, or tinidazole), probiotics, and topical mesalamine for “pouchitis” and “cuffitis.” His past medical history includes acute pancreatitis and portal vein thrombosis, but no extraintestinal manifestations of inflammatory bowel disease.

**Figure 1.**
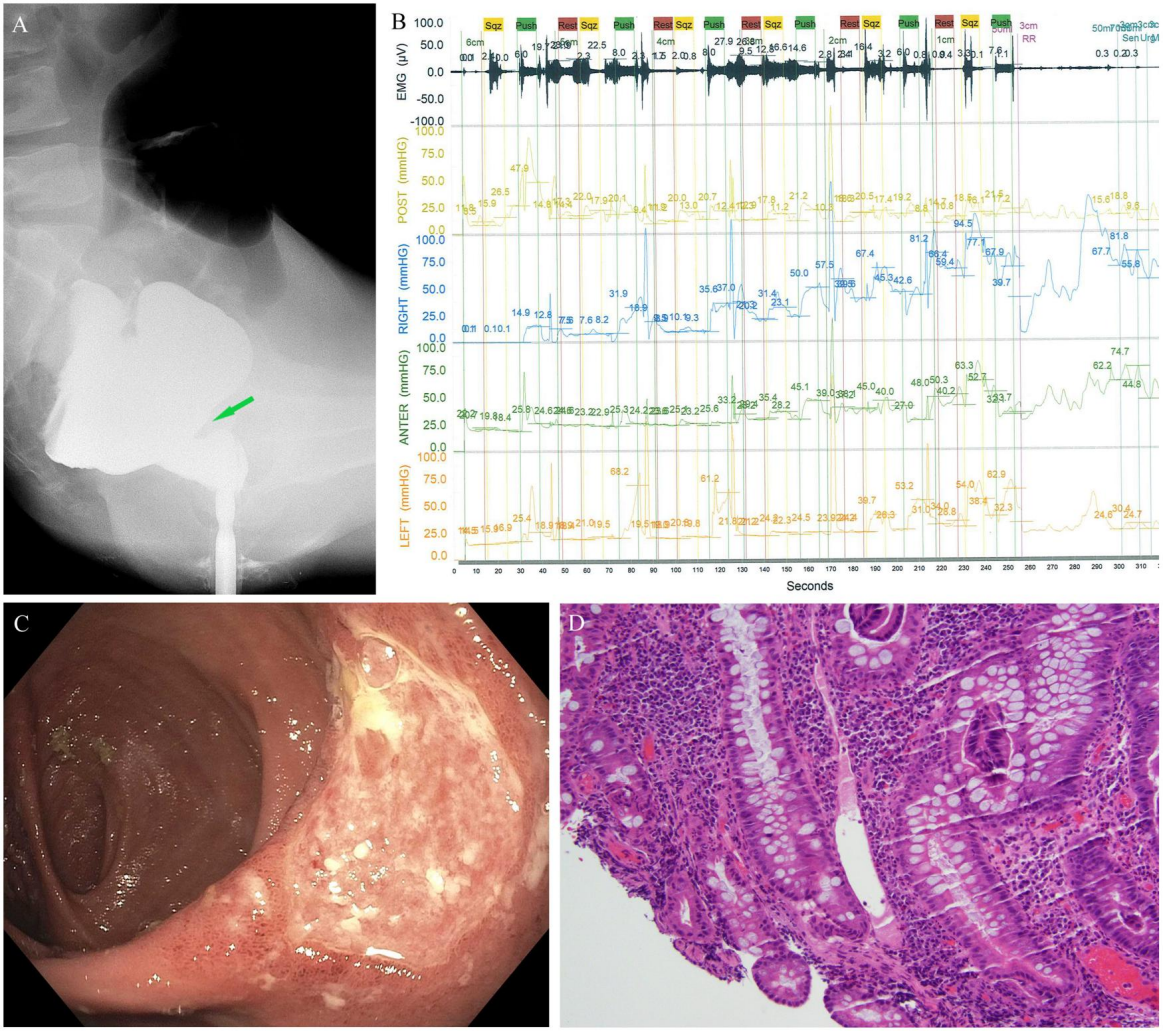
Abnormal examination findings in a patient with solitary pouch ulcer syndrome (SPUS). (**A**) Prominent distal pouch fold on barium defecography, corresponding to the location of the pouch ulcer on endoscopy. (**B**) Paradoxical contraction on manometry. (**C**) Characteristic large superficial ulcer at the distal pouch fold on pouchoscopy. (**D**) Tissue biopsy (hematoxylin and eosin stain) showed muscularization of the lamina propria and vessel dilatation.

At our Pouch Center, we performed barium defecography, which showed normal pelvic floor motions during the Kegel and Valsalva phases, but he had difficulty evacuating despite multiple attempts and prominent distal pouch fold (Figure 1A). Anopouch manometry showed slightly reduced rest and squeeze pressures, and paradox contraction with failed balloon expulsion (Figure 1B).

Pouchoscopy showed a normal prepouch ileum, erythema at the tip of the “J” area, a large 3 × 5 cm ulcerated area at the distal pouch fold of the anterior wall (Figure 1C), and a slight twist of the pouch outlet at the anastomosis. The 3-cm long cuff was normal. Tissue biopsy showed a normal prepouch ileum, chronic enteritis, severely active, with villous atrophy, crypt elongation, acute cryptitis, ulceration of pouch body, crypt architecture distortion, muscularization of the lamina propria and vessel dilatation (Figure 1D), and Paneth cell metaplasia of the cuff. He underwent 10 sessions of biofeedback therapy with improvement of symptoms but not the distal pouch fold ulcer.

## Discussion

While restorative proctocolectomy with IPAA improves patients’ health-related quality of life, structural (such as stricture, anastomotic leak, and floppy pouch complex), inflammatory (such as pouchitis and cuffitis), or functional (such as irritable pouch syndrome and dyssynergic defecation) complications often occur [2, 3]. These disease categories can coexist. For example, anastomotic stricture may be associated with fecal stasis-associated pouchitis and chronic pouchitis can concurrently be present with presacral sinus. When patients present with frequent loose stools, pouch ulcers, and favorable responses to antibiotics, they are often diagnosed as having pouchitis. Pouchitis represents a spectrum of disease phenotypes, ranging from idiopathic to secondary; from dysbiosis-associated to ischemia-associated; and from isolated inflammation in the pouch body in classic pouchitis to inflammation in both pouch body and prepouch ileum.

In our clinical practice, solitary pouch ulcer syndrome (SPUS) can be seen in patients with structural (e.g. floppy pouch complex, history of hysterectomy, bladder or uterine prolapse) or functional (e.g. pelvic dyssynergia and sawtooth contraction) pouch outlet obstruction. The patients often have associated anxiety or depression. The presence of solitary ulcers in the pouch folds with associated structural and/or functional pouch outlet obstruction may represent a unique phenotype of ileal pouch disorders, mimicking solitary rectal ulcer syndrome and similarly posing challenges in clinical management [4]. Patients can present with symptoms of chronic antibiotic-dependent pouchitis, dyschezia, bleeding, and anemia. A combined endoscopic, histologic, radiographic, and manometric evaluation is required for the diagnosis and disease monitoring [5]. Endoscopic examination often shows solitary ulcer(s) in the distal pouch fold(s). Endoscopic evaluation plays a key role in the diagnosis and differential diagnosis of pouch disorders. The accurate appreciation of the distribution of ulcer lesions helps distinction between pouchitis (e.g. diffuse pouch inflammation and ulcers of the pouch body), Crohn’s disease of the pouch (e.g. segmental distribution of inflammation and ulcer at the prepouch ileum and pouch body), and SPUS (i.e. characteristic solitary ulcer[s] at the distal pouch fold). Histology is characterized by elongated epithelium and intramucosal fibrosis. Defecography may demonstrate dilated pouch, pouchocele, pouch prolapse, pouch twist, or failed relaxation of the anal sphincters. Manometry often shows paradoxical contractions and/or sawtooth contraction patterns.

The pathophysiology of SPUS may be similar to that in solitary rectal ulcer syndrome. Clinical presentations and underlying pathogenesis of SURS and SPUS may overlap. The patients often present with excessive straining, urgency, pelvic heaviness, and bleeding. Both functional (e.g. pelvic dyssynergia) and structural outlet obstructions (e.g. prolapse, rectocele, or pouchocele) contribute to the development of the syndromes. The ulcer in both syndromes is located mainly at the anterior wall. In SPUS, the main ulcer lesion is mainly found at the anterior distal pouch fold. SPUS is likely multifactorial involving structural (such as twist and prolapse) and functional (such as dyssynergia) components along with mesenteric fat support [6]. Its management has been challenging. Our patient’s symptoms of dyschezia and frequency responded to antibiotic and biofeedback therapy. However, the pouch ulcer persisted. Attempts will be made to treat structural outlet obstructions, such as septectomy for pouch twist [7] and band ligation for distal pouch prolapse [8].

## References

[1] Tome J , Raffals LE, Pardi DS. Management of acute and chronic pouchitis. Dis Colon Rectum 2022;65:S69–76. 35905290 10.1097/DCR.0000000000002562

[2] Shen B , Kochhar GS, Kariv R et al Diagnosis and classification of ileal pouch disorders: consensus guidelines from the International Ileal Pouch Consortium. Lancet Gastroenterol Hepatol 2021;6:826–49. 34416186 10.1016/S2468-1253(21)00101-1

[3] Roussel BN , Shah SA. Diagnosis and management of functional pouch disorders: a systematic review. Dis Colon Rectum 2022;65:S113–8. 36399771 10.1097/DCR.0000000000002586

[4] Malouf AJ , Vaizey CJ, Kamm MA. Results of behavioral treatment (biofeedback) for solitary rectal ulcer syndrome. Dis Colon Rectum 2001;44:72–6. 11805566 10.1007/BF02234824

[5] Shen B. Endoscopic evaluation of the ileal pouch. Dis Colon Rectum 2024;67:S52–69. 38276962 10.1097/DCR.0000000000003269

[6] Gao XH , Khan F, Yu GY et al Lower peripouch fat area is related with increased frequency of pouch prolapse and floppy pouch complex in inflammatory bowel disease patients. Int J Colorectal Dis 2020;35:665–74. 32020266 10.1007/s00384-019-03469-x

[7] Pokala S , Shen B. Endoscopic management of obstructing pouch twist. Gastroenterol Rep (Oxf) 2022;10:goac039. 36003350 10.1093/gastro/goac039 PMC9394627

[8] Shen B. Endoscopic band ligation with hypertonic glucose cushion in the treatment of ileal pouch prolapse. Gastroenterol Rep (Oxf) 2020;9:480–2. 34733536 10.1093/gastro/goaa089 PMC8560034

